# The FIMP Medicines for Children Research Network

**DOI:** 10.1186/1824-7288-36-46

**Published:** 2010-06-30

**Authors:** Ettore Napoleone, Giuseppe Mele

**Affiliations:** 1 Indirizzo: Piazza della Vittoria 14/A, Campobasso 86100, ITALIA; 2 FIMP (Italian Paediatric Federation), Via Carlo Bartolomeo Piazza, n.30, Roma 00161, ITALIA

## Abstract

The European Paediatric Regulation (EUPR) calls for the fostering of high quality ethical research and medicinal products to be used in children. The EUPR provides the background, goals, and requirements for paediatric clinical trials. Paediatric clinical trials in children are mandatory to generate data on new drugs as well as on drugs used off-label or for unlicensed indications. The Family Paediatricians Medicines for Children Research Network (FIMP-MCRN) was established in 2003 with the aim of developing competence, infrastructure, networking and education for paediatric clinical trials. The network, consisting of twenty Paediatric Regional Networks has progressed very well and has achieved valuable improvements concerning the conduct of paediatric clinical trials. Furthermore, ad hoc training programs have incremented knowledge about clinical trials in Family Paediatrician Investigators (FPI) and have made medical professionals as well as the public aware of the need and advantages of trials in children.

## Introduction

The main role of children becomes of primary importance in social organisation and the lack of availability of medicine for children creates an important problem. This problem relates to all the children in the world, not only of developing countries but also from richer parts of the world [1]. Although many generations of paediatricians and other physicians have learned to cope with the fact that off-label or unlicensed medicines are prescribed to more than half of the children, the time has come to be sure that medicinal preparations used to treat the paediatric population should undergo a higher quality ethical research and should be appropriately authorized for use in the paediatric population [2].

Paediatricians must plan specific clinical trials and must favour experiments of medicines for children with higher protection using the right balance between risks and benefits that are implied in every experiment carried out on children considering real scientific needs. In comparison to adults, metabolic changes during childhood and adolescence determine fundamental changes which involve different dosage, administration and elimination of the medicines which require specific safety and efficacy studies [3].

To improve drug therapy in children and adolescents, the EUPR publication No 1901/2006 describes the necessity for clinical trials of drugs under development, for drugs for which changes in dosage forms or new indications are submitted for approval, and for off-patent drugs that are unlicensed or used off-label in paediatrics [4,5].

## Research and experimentation in Family Paediatrics

One of the targets of Family Paediatrics is to carry out clinical research and experimentations on paediatric medicines with the ethicality and quality needed to guarantee the broadening of knowledge and the improvement of prescription appropriateness. Authorised drugs should be used only after having the results of specific studies which evaluate the effectiveness/safety profile and dosage for paediatric age groups; pharmacology culture of paediatric age groups needs to be expanded together with the necessity of offering protection to those children who participate to clinical trials, avoiding non- necessary studies, succeeding in gathering evidence about adverse drug reactions (ADRs) and establishing correct dosage for each age group [1,3,6].

Research could and must be carried out independently by Family Paediatricians and some examples have been set thanks to the specific competence of territorial paediatrics in several areas of excellence. In some cases a "solid strategic alliance" with other characters that play a key role in the paediatric research (Universities, Hospitals, Scientific Institutes for Quality of Healthcare, Scientific Associations) should be considered and main importance should be given to a close co-operation and synergy between the areas of excellence of national paediatrics [1,3,7].

## Opportunities and challenges for Family Paediatrics research

1) Using authorised drugs only after having the results of specific studies which evaluate the effectiveness/safety profile and dosage for paediatric age groups; 2) Reducing "off-label" prescriptions; 3) Sponsoring health and prevention in paediatrics by educating and making family paediatricians be part of clinical research; 4) Improving information quality about the use of medicines for children; 5) Avoiding non-necessary clinical experimentations on children and repetition of studies; 6) Offering protection to children who participate to clinical experimentations; 7) Developing of a pharmacology culture about paediatric age groups; 8) Determining dosage for each age group in order to avoid dangerous adverse drug reactions.

The area of excellence of Family Paediatrics "Research and Investigations" has been organised by setting up a research network which has the following targets: 1) a build up of specific competence; 2) an organisation of the structure of the research area; 3) an accuracy of method and quality control of clinical trials. 4) an international activity by being part of networks of European excellence. 5) a projection towards independent research [1,3,7].

## Pharmacological experimentation, phase III and IV

Clinical research, experimentation and practice are all important aspects of the medical and paediatric professions and, as for territorial paediatrics, what is well known is that it has greater possibilities compared to hospitals and universities when safety research has to be carried out especially for some categories of medicines or for those illnesses which are not followed by hospitalisation, considering the de-hospitalisation of many pathologies, especially in paediatric age groups [1,3,7,8].

Today, unfortunately, companies are not very inclined to carry out clinical studies on medicines of paediatric use because, except for only some therapeutic categories, paediatric use represents only a small slice of the market and therefore many drugs are used inappropriately with negative safety effects in children. We also know that some ADRs can be noticed in quality (type of side effect) and quantity (real incidence in the population treated with the drug) only after the commercialisation and during the use in the "normal" population and not in those people selected for experimentation. When the drug is used in clinical practice with large numbers of non selected people, post-marketing epidemiological studies can be useful because of the feedback of all the events which occur during surveillance, with the estimate of ADR incidences which cannot be obtained through spontaneous report [3,9,10].

## Ministerial Decree n. 139/2001

Family Paediatricians can carry out phase three and four of clinical studies according to the Ministerial Decree 139/2001 which states: 1) Clinical experimentations of phase III medicines and special phase IV clinical experimentations can be carried out by Community Based Paediatricians (CBP); 2) General practitioners and CBP authorised to carry out clinical experimentations must be enrolled in a specific register which is established and updated by each Local Health Board. CBP, enrolled in specific Local Health Board registers, are authorised by the general manager of the local health board to carry out experimentations after a previous positive opinion of the Ethics Committee; 3) Public Health develops training activities which have the aim to improve the competence of CBP' clinical research, standardise procedures which lead to qualifications needed for a good clinical practice and improve Local Health Board inspections; 4) Experimentations can be carried out in all those surgeries, of both single and/or associate doctors, which have all the characteristics (logistics, instrumentations, etc.) in accordance with the protocol of studies and the principles of good clinical practice (ICH-GCP); 5) Experimenters are not allowed to have direct business relations with their sponsors; any kind of business relation must be made with the Local Health Board and the latter must draw up the measures laid down during the agreement. The experimenter's pay will be decided by the Local Health Board within the limits of its medical care plan and it could preferably consist of the development of infrastructures, service, equipment and staff; 6) Experimentation results must be made known and used for the purpose of submitting an application in case of issue or renewal of the authorisation for the commercialisation of the medicine.

## AIFA Regulation n. 76 of 31/03/2008: observational studies

In March 2008, the guidelines for observational studies were published. The key points of the Decree about Observational Studies: AIFA guidelines (Official Gazette n. 76 of 31/03/2008) are as follows: 1) the National register of observational studies has been established at AIFA and all the data referring to these studies should be sent by computer; 2) it is not necessary that General Medicine doctors and CBP should be enrolled in the registers according to the Ministerial Decree DM 139/2001 in order to carry out observational studies; 3) a formal request of approval must be made to the Ethics Committee for prospective cohort studies during which patients are followed and treated pharmacologically for any future evaluations of the results; 4) for all other types of studies a notification to the Ethics Committees of the institutes which participate is necessary and the study can be started after sixty days from the notification with a tacit consent.

Therefore, after having cast light on these regulations, we understand that all Italian Family Paediatricians can carry out epidemiological observational studies, phase IV studies, post marketing studies and surveillance studies. On the other hand, only those paediatricians (FPI) who are enrolled in the experimenters' registers (established by Local Health Boards), that is all the paediatricians who have attended and passed training courses voluntarily and that have ambulatories with necessary requirements, can carry out experimental studies.

The Ministerial Decree D.M. 139/2001 avoids any conflicts of interest by banning all business relations between sponsors and FPI as only Local Health Boards can make economical agreements, suggesting a development of infrastructures and service as payment.

## The FIMP-MRCN

The difficulties when running paediatric trials and the complexity of their methodological procedures as well as the lack of know-how when running paediatric trials and the need of resources, have all led to the decision to create the FIMP Medicines for the Children Research Network (FIMP-MCRN).

The FIMP-MCRN founded in April 2003. It has been created to improve international standards of ethics and scientific quality by means of an agreement between individuals co-operating for the same objectives, goals and quality standards. More than 6000 Italian Family Paediatricians are involved from 20 Italian Regions (every Italian Region is coordinated by a Regional Referent), covering the entire Italian territory. Until now about a thousand of these Family Paediatricians have attended training courses voluntarily and enrolled in experimenters registers, becoming FPI [8-13]. Since every paediatrician takes care of 900 children and follows them from their birth to 14 years of age, the FIMP-MCRN can survey more than 6.000.000 Italian children. Family Paediatricians (FP) are an important reference for those families with which they establish a strong and long lasting relationship based on trust [8-13].

The aims of the FIMP-MCRN are: 1) to work with the international standards of ethics and scientific quality in order to follow the planning, carrying out, registration and report of clinical investigations that are involved in paediatric subjects (Good Clinical Practice); 2) to promote the availability of safe and effective medicines for children; 3) to identify specific needs for the use of medicinal products in order to avoid duplicates of the various investigations in children and to pay attention to those that are the most biased in clinical studies; 4) to assess the efficacy of new medicines for children; 5) to survey the use of off-label and unlicensed children drugs; 6) to use orphan drugs in children; 7) to validate clinical methods for assessing the safety and efficacy of new medicines for children and also of those in use; 8) to survey adverse drug reactions (ADRs) and to promote the use of Case Report Forms [8-13].

Because of the elevated number of patients being followed, this Network is a great resource for all the investigations carried out on the use of medicines for children. The FIMP supports paediatric trials thanks to its structure which includes the FIMP-MCRN, 20 Local Research Networks, 6 Sub speciality Networks, special FIMP Working Groups and the National "Research and Investigations" Paediatric School.

The FP-Investigators took part in the majority of the most important clinical studies carried out in Italy in the last 6 years, helping in a considerable manner to create quality research and to acquire a high level of competence. In this way FIMP-MCRN guarantees continuity in time and constant cooperation between national and international networks [8-13].

Six sub-speciality networks have already been set up (vaccines, nutrition, phytotherapy, allergology, dermatology, gastroenterology) each led by a national representative and supported by other 20 regional representatives. All groups work "in synergy" with a continuous and constant exchange of information. For the past 6 years, the members of the FIMP-MCRN have had valuable experience in networking. It is obvious that networking requires, besides paediatric and trial excellence, also team spirit, team players and shared goals as well as natural trust and confidence.

The FIMP - MCRN have developed and improved expertise in: observational and epidemiological studies; long term follow up (LTFU) clinical studies; efficacy studies (DBPC-RTC and/or comparative treatment studies); safety studies (Post-marketing, Pharmacovigilance, Phase 4 clinical studies).

## FIMP Training Program

Family paediatricians interested in drug research and investigation must attend voluntarily specific training courses which are organized in all Italian regions and/or districts as established by the Ministerial Decree D.M. 139/2001 (in order to become FP- Investigators). FIMP-MCRN must, as a mandatory task, build up all the competences required, facilitate co-operation, and avoid the duplication of studies. Furthermore, FIMP-MCRN aims to organize a higher standard of training, creating a Clinical Research Master Course and an FIMP-National School of Research and Investigations which has no other object than to train all the FPI according to the principles of paediatric clinical trials.

Creating specific competence means:1) creating a paediatric "expertise" which will be necessary for better quality research. 2) implementing and wide-spreading the FIMP-MCRN which already exists on a national scale. This can happen thanks to two separate levels of training: the first level with the training of a large number of FPI (M.D. n.139/2001); the second level with the training of a team of FPI (by means of Masters and Research Schools) who will have a greater competence and will have to guarantee the quality of clinical studies [8-13].

For this purpose, training courses for FPI were run. The main goals of the courses were: 1) to establish experimenter's responsibility, ethical regulations and information quality to be given to patients. 2) to help Paediatricians acquire all technical and cultural know-how in order to guarantee an independent carrying out of a research plan according to ethical principles, without changing its professional profile. 3) to determine the most adequate methods of developing a rigorous safety examination activity and an appropriate use of medicines in paediatrics. The main themes examined are: a) the importance of clinical research and drug experimentation in paediatrics; b) the distinctive feature of pharmacokinetics and pharmacodynamics in paediatric age groups, the regulations, the terminology and methodology of experimentation; c) a functional use of pharmacological therapy in order to reveal the best risk/benefit balance; d) a Good Clinical Practice, guidelines and ethical comments about medicine experimentation in paediatrics; e) the structure of a research project keeping in mind ethicality, criticality and quality; f) the role and responsibilities of the Ethics Committee, the relationships between researchers-sponsors-monitors-CE, informed consent and approval of minors, medical-legal and insurance aspects, privacy, aspects of clinical epidemiology and healthcare statistics; g) the evaluation and taking care of unfavourable events during clinical experimentation of medicines and with particular reference to Pharmacovigilance on the territory; h) the opportunities and criticality of family paediatricians' research and experimentation.

A Research School will be founded, which will also be a pro-active observatory of analysable and improvable bio-psychosocial phenomena. These will then help, in scientific terms, Family Paediatricians to acquire the necessary competence for "high quality research standards" directed to the safeguard of vulnerable people such as paediatric age group subjects.

At the end of the training, FPI will be able to plan and run a research protocol; they will also be able to apply all the ethical regulations together with all the methodologies and strategies for a scientific autonomy in order to improve their own competence and medical care standards. A category of FPI, who will be experts in running clinical studies and with tested abilities, will be the core of all of the scientific production when talking about territorial paediatrics.

## FIMP Quality Management System

The first step towards structural organisation was taken when the AIFA, as an institution, awarded the recognition of Institute of FIMP Excellence in Research and Investigations to a non-profit scientific or research association/company. The above mentioned institute is the FIMP-MCRN. This Network has been acknowledged as a National, transversal network by the EMA.

A clinical research project seeks the cooperation of a large number of people with different types of competence and who must work according to precise methodological rules in order to assure high standards of quality. Many other characters support family paediatrician clinical studies (secretaries, administrative workers, accountants, experts in policy privacy, computer engineers, etc.) in order to organise perfectly structured trials which avoid family embarrassment and concern.

The FIMP has got a quality management system, a Quality Control System (FPI- Monitor) that guarantees the ICH-GCP, a Quality Assurance (FPI- Auditor) which carries out some audits to check the QA as required by the ICH regulations, a Legal and Insurance advise is given for both forensic medicine and insurance aspects of trials and for policy privacy, an Fimp in-house Steering Committee (a preliminary control before sending the research projects to the National Ethical Committees), a Safety Monitoring (control of possible ADRs in clinical trials). The traceability, the transparency and the safety of data are submitted to an computer engineering company called VAM Srl which has an ISO 27001 Certification.

The role of the Quality Assurance (FPI-Auditor) therefore, is not "to produce" quality but to verify that all the different aspects of the system are satisfactory. Audits are carried out in accordance with the European Network of GCP Auditors and other GCP Experts (ENGAGE).

FIMP has improved its own standards of operational procedures (SOP), granting the highest levels of control in transparency, safety and quality of data and of submitted studies. Statistic analysis is carried out by both statistic experts and FPI with expertise in healthcare statistics. The Coordination Centre has the task to monitor the Enrolling Centres, the observance of GCP regulations and a surveillance task on the follow ups. Secretarial staff arranges investigator's meetings, are a logistical connection and support with the enrolling centres and Ethics Committees.

## Research Working Group (RWG) and Steering Committee

Under the supervision of the head of FIMP-MCRN and with the collaboration of Regional Referents a Research Working Group (RWG) has been formed with the following aims: 1) establish territorial Inventories of Paediatric Needs which can help to understand research priorities; 2) monitor off-label medicines which disadvantage the paediatric population; 3) monitor medicines which are close to patent expiry; 4) monitor orphan drugs (rare diseases); 5) to urge towards a iatrogenic pathology culture which creates a more careful control of Paediatric medicines' risk/benefit ratio; 6) create an Network of pharmacovigilance which can work in synergy with the National Network of Pharmacovigilance;7) establish territorial surveys (e.g. Medicine Registers).

This Group will be called to draw up research projects especially for independent research (AIFA notices for a competition, Ministerial notices for a competition, etc).

The Paediatric Working Group is coordinated by a Steering Committee inside the FIMP, formed by family paediatricians who have the best competence and experience. The Steering Committee, with the responsibility and supervision of the Head of FIMP-MCRN, will have the task of proposing research projects, regular reviewing, organising training courses, epidemiological investigating, attending to international conferences, magazine publishing with IF and everything which has to do with science and that will be considered useful for family paediatricians' clinical research. Moreover, the Steering Committee will control, verify and implement all the scientific activities proposed and/or suggested by each member of the RWG.

Finally, the Steering Committee will have the task to verify the methodological quality of all the research projects which will then be evaluated by Ethics Committees.

## Clinical studies experience

Since 2003, the year FIMP-MCRN was founded, Family Paediatricians have participated to various clinical studies and research projects in collaboration with the most important national Universities and/or Research Institutes [14-26]. This has allowed them to acquire experience and rigorous methodology in clinical paediatrics research. Despite the fact that in the opening phases, Family Paediatrician involvement in clinical trials was quite poor throughout the Italian territory, from 2007 a national organisation has created the characteristics for a fully independent scientific running of clinical trials [27].

This involved the best Family Paediatricians and Regional Referents together with so many other professionals, each with their own competence (lawyers, statistic experts, secretaries, computer engineers, etc.) which has assured quality, methodology and a correct running of clinical studies.

The objectives of the FIMP - MCRN are to develop expertise in multi-centre clinical drug trials in the paediatric population. The network is coordinated by the Head who is responsible for organizing and performing certified training programs and network meetings, feasibility assessments, communication with companies, Ethics Committees, and regulatory authorities (AIFA-Italian Agency of Drug Evaluations).

Harmonized Standard Operating Procedures (SOP), a clinical trial database, standardized essential study documents (e.g. patient and parent information, informed consent, documents for assent), and a curriculum for training courses for FPI, have all been established.

A great part of completed trials since 2003 are academia based studies. From 2009 there are four on going clinical studies. The majority address drugs in the following areas: gastroenterology, allergology, dermathology, neurology, infectious diseases and disease prevention. The FIMP - MCRN also share competence and experience with other European networks.

## Epidemiological post-marketing studies

When the drug is used in clinical practice in large unselected populations, epidemiological post-marketing studies are useful as they find their major confirmation in recalling all the events that occur during monitoring, with estimates of incidence of ADRs that can not be obtained by spontaneous reports [3,9,10]. In these studies a significant role can be played by the FPI with the participation to active pharmacovigilance projects:

### 1) Family Paediatricians -FIMP- Antibiotics Tolerability Profile Study (FIMP-ATPS)

The FIMP wanted to promote a study of the assessment of tolerability in the days of administration of Cefaclor compared with amoxicillin-clavulanate [27].

The primary objective of the Study (FIMP-ATPS) was to assess in patients with pharyngotonsillitis (FT) and rhinosinusitis (RS) the tolerability profile of Cefaclor and Amoxicillin + Clavulanic acid after the days of administration. For the group of patients assigned to treatment arm the regime of Cefaclor dosage was 50 mg/kg/day for 5 days for the FT and for 14 days for the RS, for the one with amoxicillin + Ac. Clavulanic it was 50 mg/kg/day for 10 days for the FT and for 14 days for R.S. A total of 537 patients of both sexes, between 4 and 14 years of age were enrolled, including 435 diagnosed with pharyngotonsillitis (FT) and 102 cases with a rhinosinusitis (RS) diagnosis.

No major reactions were reported. Reported minor adverse reactions were: nausea, vomiting, diarrhea, abdominal pain, rashes, itching, loss of appetite, bloating, borborygmi, constipation, change in the hive, irritability.

In the Cefaclor-group (257 children): 26 children (10,1%) have reported only one minor reaction, 41 (16%) more than one minor reaction and 190 (73,9%) did not report any reactions.

In the Amoxicillin-Clavulanic Acid group (262 children), 37 children (14,1%) have reported only one minor reaction and 71 (27,1%) more than one minor reaction, while 154 (58,8%) did not report any reactions. Minor adverse reactions, mainly gastrointestinal plead for a better Cefaclor tolerability (p < 0.001) when comparing Cefaclor to Amoxi-clavulanate.

### 2) (H1N1) Flu virus Surveillance System - INFLUNET Family Paediatricians Sentinel in collaboration with ISS (National Institute of Health)

Starting from 19 October 2009, the H1N1 Flu Virus surveillance system is based on Influnet and collects all the cases reported of those clients of the network of sentinel physicians, as well as data on viruses circulating the network of accredited laboratories.

In epiweek 45 [28] the incidence of H1N1, as detected through sentinel FP surveillance, was 28.19 per 1,000 in the 0-4 yrs. age group and 40.78 per 1,000 in the 5-14yr age group After having peeked the H1N1 epicurve has declined and then stabilized.

1172 reports of suspected adverse reactions after the administration of pandemic vaccine Focetria, out of a total of 898.562 doses were included in the National Pharmacovigilance Network.

About 15% reports are of children up to 11 years of age (7 severe) and 4% relate to teenagers and all other older age groups. Most frequently reported reactions are headache (the most frequently reported reaction of the nervous system), fever and joint pain. At the moment of administration various types of local reactions such as pain, swelling, erythema were also reported [29].

### 3) Disease Register -FIMP DUMBO Otitis Study

The study is an epidemiological survey on observed acute otitis media in a Italian pediatric population. The main objective of the project is to create a disease register and to evaluate the incidence of AOM in all children aged between 0 and 59 months in relation to their clinical severity or recurrence. The clinical diagnosis of AOM, according to the recommendations of the AAP, will be supported by a Pneumatic Otoscope. This study is on-going

## International activity of FIMP-MCRN

The European Paediatric Regulation have the aim to increase the number of suitably studied medicines adapted for a potential use in children of different age groups and to go towards research on an international basis by planning to establish, thanks to EMA's help, a European Network of researchers and institutes with specific competence in running pharmacological experiments in the paediatric field [4,5].

To achieve these objectives the EMA is tasked with developing a European Network of Paediatric Research at the EMA (ENPREMA) of existing national and European networks and centres with specific expertise in research and clinical trials relating to paediatric medicines with the scientific support of the Pediatric Committee. The ENPREMA objectives will be: a) to harmonize clinical trial procedures (SOP's, documents, data management, monitoring, training curricula, etc.) - at European level; b) to define strategies in order to solve major challenges of pediatric clinical trials (insurance, divergent Ethic Committee positions, lack of pediatric expertise, etc.) - at European level; c) to ensure effective coordination and communication between individual networks, investigators and pediatric clinical trial centres; d) to stimulate and facilitate the development and integration of new national networks and trial centres; e) to link together existing networks; f) to provide expertise and access to infrastructure for industry to conduct studies in children; g) to define consistent and transparent quality standards; h) to define strategies for resolving major challenges; i) to communicate with external stakeholders.

On the 16th of February 2009 the EMA organised a one-day workshop to discuss and initiate the development of this ENPREMA [6]. The result of this workshop was the proposal to create a Coordinating Group and to point out recognition criteria and quality standard to be fulfilled by networks which intend to become members of the future ENPREMA.

The objectives Coordinating Group will be: 1) to contribute to the short and long-term strategy; 2) to discuss and solve operational and scientific issues; 3) to discuss and confirm scientific quality standards; 4) to act as a forum for communication; 5) to contribute to the short and long-term strategy of the network; 6) to report to the Pediatric Committee (PDCO).

Two working groups were formed: the first with the task to elaborate structure, composition and duties of the Coordinating group, the second with the task to elaborate and find agreement consensus on the recognition criteria and quality standard.

On the 16th of March 2010 the EMA organised a second one-day workshop to discuss the right composition of the Coordinating Group and the publication of finalised recognition criteria for self-assessment.

Even Family Paediatrics, thanks to its Network, recognised at a European level (can be found in the list of European Networks), is ready to become part of the European Network by helping to create the ENPREMA.

Family Paediatrics is part of the Working Group of Medicines for Children of the EAP (European Academy of Paediatrics) and this will grant new medicine research opportunities in the paediatric field and will guarantee an enduring of experience and competence.

In 2010 the FIMP- MCRN has become a member of the ENCePP (The European Network of Centres for Pharmacoepidemiology and Pharmacovigilance).

The FIMP-MCRN participated in international conferences last year (Excellences in Paediatrics -Florence) presenting the results of epidemiological pharmacovigilance surveys [30] and will participate this year for other International Congress (IPA- Johannesburg and EAPS-Copenhagen).

## Future prospects

Paediatric clinical trials are necessary to make progress in children's care. The EUPR wants to link together and coordinate existing networks, establish a platform for communication and exchange of information between European networks and share the skills and expertise in order to cut to shape and influence future development in paediatric research. Networking is necessary to build up the competence, to make co-operation easier and to avoid duplication of studies.

The main target of Family Paediatricians should be to regain possession of the research aspect of the profession through the fundamentals of science and the production and publishing of paper work about primary care in order to guarantee greater knowledge and better decision-making.

The net must be aware and ready for specific training so that it can answer with authority for independent research. Quality and ethicality of research are becoming important end-points of territorial Paediatrics, opportunities and challenges of Family Paediatricians during their education and during the sponsoring of health and prevention in the paediatric age groups.

Family Paediatricians are convinced that the path they are following is the right one and this is due to the fact that they have great scientific enthusiasm even if they understand that a long distance still has to be covered and there is so much to do in order to make territorial paediatric research possible and useful.

One of the chances in the future is to become part of the ENPREMA which carry out pharmacological experiments in the paediatric field and another one is to take part in pharmacovigilance research coordinated by ENCePP.

These possibilities could grant new paediatric medicine research opportunities for Italian family paediatricians and could guarantee further exchange of experience and competence, being an important wedge in the cultural mosaic of paediatric research.

## Competing interests

The authors declare that they have no competing interests.

## Authors' contributions

EN conceived of and drafted the manuscript. GM participated in its design and in its coordination. The two authors read and approved the final manuscript.

## Author's list

Ettore Napoleone is the Head of FIMP- MCRN (Family Paediatricians -Medicines for Children Research Network), Member of Paediatric Working Group AIFA (Italian Medicines Agency) and President S.I.P (Italian Society of Paediatrics) - Molise

Giuseppe Mele is the President of FIMP (Italian Paediatric Federation)
